# The Prognostic PDE4D7 Score in a Diagnostic Biopsy Prostate Cancer Patient Cohort with Longitudinal Biological Outcomes

**DOI:** 10.1155/2018/5821616

**Published:** 2018-07-26

**Authors:** Dianne van Strijp, Christiane de Witz, Pieter C. Vos, Eveline den Biezen-Timmermans, Anne van Brussel, Janneke Wrobel, George S. Baillie, Pierre Tennstedt, Thorsten Schlomm, Birthe Heitkötter, Sebastian Huss, Martin Bögemann, Miles D. Houslay, Chris Bangma, Axel Semjonow, Ralf Hoffmann

**Affiliations:** ^1^Philips Research Europe, High Tech Campus 34, 5656AE Eindhoven, Netherlands; ^2^Institute of Cardiovascular and Medical Science, University of Glasgow, G12 8TA Glasgow, Scotland, UK; ^3^Martini-Klinik Prostate Cancer Center, University Medical Center Hamburg-Eppendorf, 20246 Hamburg, Germany; ^4^Klinik für Urologie, Charité–Universitätsmedizin Berlin, 10117 Berlin, Germany; ^5^Gerhard-Domagk-Institute of Pathology, University Hospital Münster, 48149 Münster, Germany; ^6^Prostate Center, University Hospital Münster, 48149 Münster, Germany; ^7^Institute of Pharmaceutical Science, King's College London, WC2R 2LS London, UK; ^8^Mironid Ltd, BioCity Scotland, ML1 5UH Newhouse, Scotland, UK; ^9^Department of Urology, 3000CA Erasmus Medical Center, Rotterdam, Netherlands

## Abstract

*Purpose. *To further validate the prognostic power of the biomarker PDE4D7, we investigated the correlation of PDE4D7 scores adjusted for presurgical clinical variables with longitudinal postsurgical biological outcomes.* Methods. *RNA was extracted from biopsy punches of resected tumors (550 patients; RP cohort) and diagnostic needle biopsies (168 patients; DB cohort). Cox regression and survival were applied to correlate PDE4D7 scores with patient outcomes. Logistic regression was used to combine the clinical CAPRA score with PDE4D7.* Results. *In univariate analysis, the PDE4D7 score was significantly associated with PSA recurrence after prostatectomy in both studied patient cohorts' analysis (HR 0.53; 95% CI 0.41-0.67; p<1.0E-04 and HR 0.47; 95% CI 0.33-0.65; p<1.0E-04, respectively). After adjustment for the presurgical clinical variables preoperative PSA, PSA density, biopsy Gleason, clinical stage, percentage tumor in the biopsy (data only available for RP cohort), and percentage of positive biopsies, the HR was 0.49 (95% CI 0.38-0.64; p<1.0E-04) and 0.43 (95% CI 0.29-0.63; p<1.0E-04), respectively. The addition of the PDE4D7 to the clinical CAPRA score increased the AUC by 5% over the CAPRA score alone (0.82 versus 0.77; p=0.004). This combination model stratified 14.6% patients of the DB cohort to no risk of biochemical relapse (NPV 100%) over a follow-up period of up to 15 years.* Conclusions. *The PDE4D7 score provides independent risk information for pretreatment risk stratification. Combining CAPRA with PDE4D7 scores significantly improved the clinical risk stratification before surgery.

## 1. Introduction

Prostate cancer displays as a heterogeneous disease with varying potential to develop progressively to deadly forms of the disease [1]. Clinically, various schemes for pretreatment risk classification have been developed based upon longitudinal biological patient outcomes [2]. While active surveillance (AS) is recommended by the various guidelines for men with very low and low risk prostate cancer [3], there is a significant subgroup in this patient population with a risk of 10-25% cancer recurrence after primary treatment [4–6]. These patients suffer from the burden of follow-up treatments that are typically triggered by biochemical relapse. Likewise, in the intermediate risk group, there is a subpopulation with low risk of biochemical progression [7, 8]. Nevertheless, this group is heterogeneous, comprising patients with varied outcomes, including those with aggressive pathological characteristics [9, 10].

Clinical risk descriptors do not delineate effectively either the extent of the disease or its aggressiveness for all patients. Additional molecular information representing the biology of the disease offers the potential to achieve this.

Cyclic AMP is an ubiquitous second messenger that critically impacts on the functioning of all cell types in the body through its effectors, protein kinase A (PKA) and Exchange Protein Activated by cAMP (Epac) [11, 12]. It has the ability to regulate independently distinct processes within a single cell type due to compartmentalisation of signalling processes to spatially discrete loci (signalosomes) [13, 14]. This is achieved not only by the sequestration of effector molecules and their targets, but also by the formation and shaping of intracellular gradients of cyclic AMP by its targeted degradation through the action of cAMP degrading phosphodiesterases (PDEs) that are specifically sequestered to distinct signalosomes in discrete cell locales [14]. Playing a critical role in this are members of the cAMP specific PDE4 family. Indeed, this four-gene family (PDE4A/B/C/D), which encodes 20+ isoforms, appears to be designed specifically to underpin compartmentalisation as their isoform-specific N-terminal regions contain motifs that allow for their targeting to distinct signalling complexes [15]. Consistent with the importance of particular PDE4 isoforms, their selective knockdown leads to distinct phenotypic changes [16] and genetic lesions in particular PDE4 subfamilies have been shown to provide the underpinning molecular pathology [17]. PDE4D7 is the so-called long isoform as it contains both the UCR1 and UCR2 regulatory domains that allow for regulation by various protein kinases, including PKA and MK2 as well as determining the functional outcome of catalytic unit phosphorylation by ERK [14]. Functionally, then it contributes to the cellular desensitization system towards cAMP and enables cross-talk between signalling pathways that lead to the activation of ERK and AMPK.

Recently, we have demonstrated that PDE4D7 transcript levels correlate to the longitudinal outcome of prostate cancer and independently add to postsurgical risk stratification in a consecutively operated prostate cancer patient cohort [18, 19]. To further explore the prognostic power of PDE4D7 in pretreatment patient risk assessment, we have investigated here the correlation to disease recurrence in the context of presurgical risk variables and algorithms like the CAPRA (Cancer of the Prostate Risk Assessment) score. We have developed a combination model of CAPRA and PDE4D7 scores in a surgery cohort and validated this model in an independent patient cohort on diagnostic biopsy tissue. We conclude that PDE4D7 does not only provide value in postsurgical risk stratification as we have shown before but also increases the stratification power of the CAPRA score in a pretreatment setting.

## 2. Patients and Methods

### 2.1. Patient Cohorts and Samples

RP patient cohort (n=550): patients consecutively managed at a single, large-volume prostate cancer center were included into the study (Martini-Klinik, Hamburg, Germany). Two small biopsy punches (~1x2 mm) of a representative resected tumor area of patients operated on between 2000 and 2004 were collected from the tumors index lesion. Patients who underwent adjuvant hormone therapy were removed from the study cohort. RP∗ patient cohort (n=130): detailed characteristics of this cohort and analysis of the respective gene expression data were described previously [20].   DB patient cohort (n=168): from the tumor positive diagnostic biopsy with the highest Gleason grade per patient a single biopsy punch (~1x2 mm) was collected. Patients were diagnosed with prostate cancer and operated on between 1994 and 2011 at the Prostate Center (University Hospital Münster, Germany). After quality control of the study data based on predefined criteria [18] 503 and 151 patient samples were defined eligible for statistical analysis in the RP and the DB cohort, respectively. The local Institutional Review Boards approved the collection of patient tissue for clinical research with appropriate patient consent (for cohort design see Supplementary Figures 3A & 3B).

### 2.2. Laboratory Methods

To account for potential tumor heterogeneity, the two tissue punches of the RP cohort were combined before nucleic acid extraction. A potential difference in tumor cellularity of the tissue punches was addressed by normalization of the qPCR results of the PDE4D transcript to four reference genes which were selected based on stable gene expression across multiple tumor sample types [18]. All used molecular laboratory methods including oligonucleotide primers and probes for RT-qPCR (quantitative real-time PCR), RNA extraction, and quality control and procedures to include/discard samples from the statistical analysis were described before [18].

### 2.3. Data Analysis and Statistics

Generation of normalized PDE4D transcript expression was performed by subtracting the Cq of the respective PDE4D transcript from the averaged Cq of the reference genes. Normalized PDE4D7 expression was transformed to the PDE4D7 score [19] (Supplementary Figures 1A & 1B). In correlation analysis for various available biological and treatment related outcomes (Table 1), the PDE4D7 score was used either as a continuous or as a categorical variable defined as (a) PDE4D7 score (1≤2); (b) PDE4D7 score (>2 and ≤3); (c) PDE4D7 score (>3 and ≤4); (d) PDE4D7 score (>4 and ≤5). The CAPRA risk score and corresponding low (1), intermediate (2), and high risk (3) categories were calculated as described earlier [21]. Uni- and multivariate Cox regression and Kaplan-Meier analyses were applied to correlate biochemical recurrence (BCR) progression-free survival or secondary treatment (salvage radiation and/or androgen deprivation) free survival (STFS) to the PDE4D7 score in the RP cohorts (n=503 [18], and Taylor et al. [20] (n=130)) and the DB cohort (n=151; this study). Decision curve analyses were performed as described [22]. For statistical analysis, the software package MedCalc (MedCalc Software BVBA, Ostend, Belgium) was used. The data analysis strategy is outlined in Supplementary Figure 2.

## 3. Results

### 3.1. Correlation of PDE4D7 Score to Longitudinal Clinical Outcomes

We recently described the association of the expression of the prostate cancer biomarker PDE4D7 to postsurgical biochemical relapse based on the quantitation of the PDE4D transcript in surgical resection tissues [18]. Here we set out to translate this earlier finding to a presurgical setting using three independent patient cohorts. Firstly, we evaluated a radical prostatectomy (RP) cohort comprising 550 patients (Supplementary Figure 3A) [18]. In this patient cohort, a logistic regression model of the PDE4D7 score together with the presurgical CAPRA risk score was developed to predict the risk of postsurgical BCR. Secondly, we tested this PDE4D7 & CAPRA risk model for BCR outcome in an independent surgery cohort (RP*∗*; n=130 [20]). It is well known that >30% of tumors with an initial biopsy Gleason 3+3 undergo grade migration to a pathology Gleason 3+4 after surgery; we could confirm this effect in the RP cohort used in this study while any other stage migration with initial biopsy Gleason scores >3+3 was much less frequent (<5%). However, we could not find a significant PDE4D7 expression difference in tumors that were upgraded postsurgically from a biopsy Gleason 3+3 to a pathology Gleason 3+4 versus those tumors that remained Gleason 3+3 after radical prostatectomy (data not shown). Therefore, we hypothesized that the association of PDE4D7 measured on resection tissue with presurgical clinical variables will translate into equivalent results on preoperatively prostate needle biopsy tissue. To test this hypothesis, we validated the PDE4D7 & CAPRA risk model to predict postoperative BCR which we developed using the RP cohort in a presurgical needle biopsy (DB) cohort. This cohort comprised 168 patients of which 151 were eligible for subsequent data analysis (Table 1; Supplementary Figure 3B).

Univariate Cox regression analysis demonstrated a very significant association of the continuous PDE4D7 score to time to BCR in the RP and DB patient cohorts with HR=0.53; 95% CI=0.41-0.67; p<0.0001, and HR=0.47; 95% CI=0.33-0.65, p=<0.0001, respectively. Adjusting the multivariate Cox regression analysis for the presurgical variables age, preoperative PSA, PSA density, biopsy Gleason grade group, percent tumor positive biopsy cores, clinical stage, or the prognostic CAPRA score [21] resulted in a significant independent contribution to the prediction of postsurgical BCR for the continuous PDE4D7 score (Tables 2(a), 2(b)).

### 3.2. Outcome Modeling of Combined CAPRA and PDE4D7 Scores

To explore this further we set out to test the benefit of a combination of the PDE4D7 score with clinical variables used to predict postsurgical risk of disease progression. Based on our multivariate Cox regression data we hypothesized that a combination of the CAPRA score, together with the PDE4D7 score, might provide a significant improvement in prognostic power over the CAPRA score alone. The CAPRA algorithm combines age, preoperative PSA, biopsy Gleason, and percent of tumor positive biopsy cores. The resulting score categorized patients into low risk (CAPRA scores 0-2), intermediate risk (CAPRA scores 3-5), and high risk (CAPRA scores 6-10). The CAPRA score has been shown to predict, significantly, postsurgical PSA relapse in several studies [23]. To evaluate our postulation we selected a subcohort of 449 patients (92 events; 20.5%) of the RP cohort with complete 5-year outcome histories and generated a logistic regression model to combine PDE4D7 with the CAPRA score so as to predict the 5-year risk of biochemical recurrence after surgery (odds ratio 0.46; 95% CI 0.3-0.69; p=0.0002; data not shown). Next, we tested this CAPRA & PDE4D7 score logistic regression model on 130 patients from the independent RP*∗* cohort [20] as well as on the 151 patients of the DB cohort for Kaplan-Meier survival analysis and ROC curve analysis, as well as decision curve analysis (see Supplementary Figure 2 for data analysis strategy).

**Figure 1:**
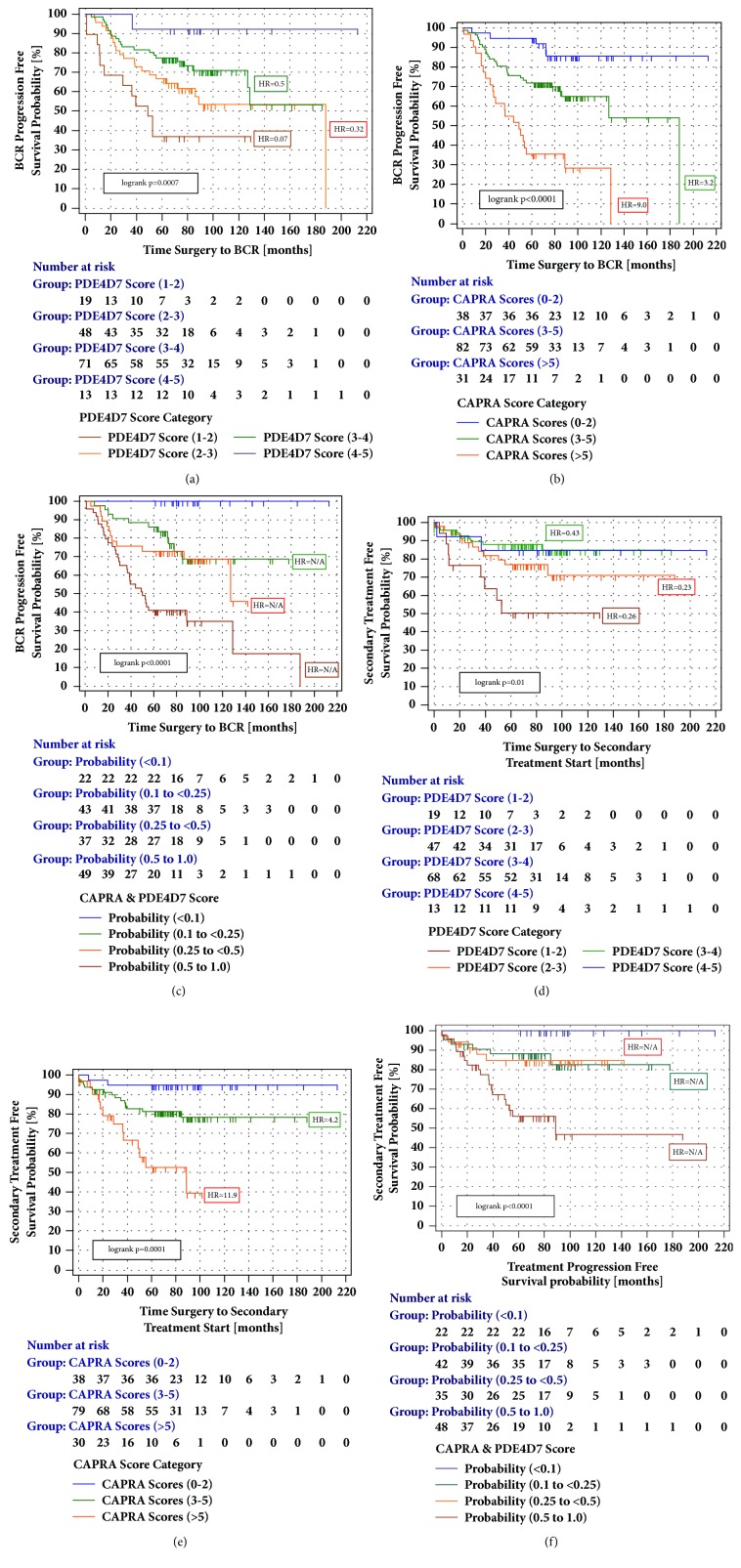
Kaplan-Meier survival analysis of the time to PSA relapse (endpoint: BCR) or start of any salvage therapy after radical prostatectomy (endpoint: STFS) in the DB patient cohort (n=151).** ((a)-(c))** Kaplan-Meier analysis of the biochemical recurrence (BCR) free survival in the patient diagnostic biopsy (DB) cohort of the categorized PDE4D7 score, the CAPRA score categories, and the CAPRA & PDE4D7 score combination model.** ((d)-(f))** Kaplan-Meier analysis of postsurgical secondary treatment free survival (STFS) in the patient diagnostic biopsy (DB) cohort of the categorized PDE4D7 score, the CAPRA score categories, and the CAPRA & PDE4D7 score combination model. The CAPRA & PDE4D7 combination model was developed by logistic regression in the RP patient cohort and used as such for testing in the DB patient cohort. Censored patients are indicated by vertical bars. PDE4D7 score categories were defined as PDE4D7 (1-2): PDE4D7 scores (1 to <2); PDE4D7 (2-3): PDE4D7 scores (2 to <3); PDE4D7 (3-4): PDE4D7 scores (3 to <4); PDE4D7 (4-5): PDE4D7 scores (4 to <=5). The CAPRA score categories were defined as CAPRA (1): CAPRA scores 0-2; CAPRA (2): CAPRA scores 3-5; CAPRA (3): CAPRA scores ≥6. The CAPRA & PDE4D7 score categories were defined according to the probability to experience PSA failure after surgery based on the logit(p) function of the logistic regression model. Four categories of probabilities were selected: p<0.1; p=0.1 to <0.25; p=0.25 to <0.5; p=0.5 to 1.0 to risk categorize the DB patients.

In Kaplan-Meier survival studies on the DB patient cohort, we observed a significant separation of patients into different risks to experience postoperative PSA relapse based on the presurgical measurement of the PDE4D7 score in a tissue punch of a diagnostic needle biopsy (Figure 1(a)). We then compared postsurgical progression-free survival curves of the three CAPRA score categories (low, intermediate, and high risk) with progression-free survival based on the CAPRA & PDE4D7 logistic regression combination model in the DB (n=151) patient cohort. Concerning the logistic regression model we categorized the model-calculated risk probability to experience the clinical endpoint into four groups (p<0.1; p=0.1 to <0.25; p=0.25 to <0.5; p=0.5 to 1.0) and compared the hazard ratio (HR) difference between the lowest versus highest recurrence risk groups between the tested models (Figures 1(b) and 1(c)). The addition of the PDE4D7 score to the clinical CAPRA categories proved to significantly increase in the hazard ratio difference from the lowest to the highest risk group in the DB cohort (HR=9.0 for the CAPRA score; HR for the CAPRA & PDE4D7 combination score could not be determined due to no event in the lowest risk group which is used as the reference; Figures 1(b) and 1(c)).

We then set out to test the clinically relevant endpoint of secondary treatment free survival (STFS). This endpoint comprises start of any salvage therapy (radiation, hormone ablation) after postsurgical PSA failure. As before, we noticed an improved risk separation between patients with lowest versus highest risk groups to receive postsurgical secondary treatment when combining the CAPRA with PDE4D7 scores versus CAPRA score categories alone (Figures 1(d)–1(f)). Finally, we confirmed the performance of the CAPRA & PDE4D7 combination model versus the CAPRA score categories on the RP*∗* cohort (HR=11.8 for CAPRA score versus HR=16.4 for the CAPRA & PDE4D7 combination score, respectively (Supplementary Figures 4A & 4B)).

**Figure 2:**
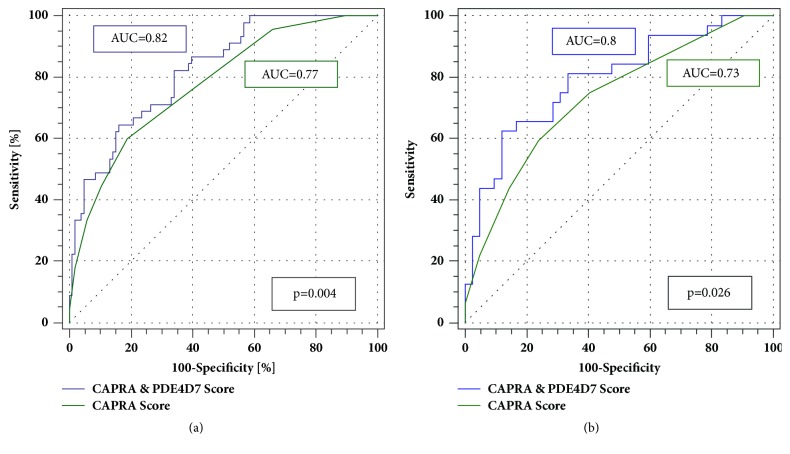
**(a)** ROC curve analysis of 5-year biochemical recurrence (BCR) in the DB cohort (n=151) of the CAPRAP score versus the CAPRA & PDE4D7 logistic regression model which was developed on the RP patient cohort with complete 5-year follow-up (n=449).** (b)** Subcohort ROC curve analysis of the 5-year biochemical recurrence (BCR) of CAPRA & PDE4D7 logistic regression model compared to the CAPRA model alone. In this analysis, only patients with a biopsy Gleason ≥7 were included (n=74).

Next, we analysed the prediction of risk of 5-year PSA relapse after primary treatment using the CAPRA score alone versus the CAPRA & PDE4D7 score combination model, which was previously developed using the RP cohort. In ROC analysis, we calculated the 5-year BCR AUCs as 0.77 for the CAPRA score alone and 0.82 for the combination model of CAPRA & PDE4D7 score model (p=0.004; Figure 2(a)). Further, the CAPRA & PDE4D7 combination model showed equivalent performance in a subcohort analysis including only patients with biopsy Gleason ≥7 over CAPRA score alone (AUC=0.8 versus 0.73, respectively; Figure 2(b); p=0.026). This data may indicate an improved discrimination performance of the combination model in patients with higher risk characteristics.

**Figure 3:**
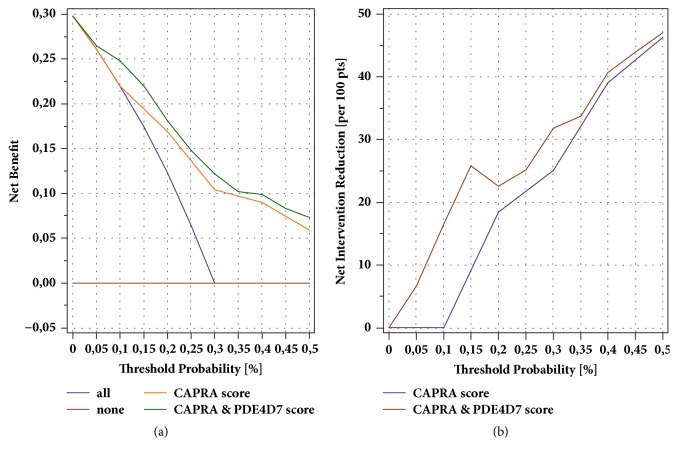
**(a) **Decision curve analysis in the diagnostic biopsy (DB) patient cohort of the net benefit of four different treatment decision strategies for men at risk of disease recurrence within 5 years after surgery. In total 45 of the 151 investigated patients failed the initial primary treatment of surgery by PSA recurrence (29.8%) within 5 years after intervention. Treatment strategies were tested for their net benefit across indicated threshold probabilities (0.05 step size) to trigger prostate surgery based on the probability of future disease recurrence. The CAPRA scores and the CAPRA & PDE4D7 scores were converted into 5-year BCR probabilities with logistic regression on the RP cohort (n=449 men with completed 5-year follow-up) before estimating net benefit.** (b)** Net reduction analyses demonstrating in how many patients a resection can be avoided based on the predicted risk of BCR derived from the CAPRA score and CAPRA & PDE4D7 score model, respectively.

Recently, the concept of decision curve analysis (DCA) was introduced to test the value of a biomarker or prediction model in clinical practice [24]. DCA is a net benefit analysis, which compares the true-positive to the weighted false-positive rates across different risk thresholds. We explored the net benefit of avoiding primary treatment based on the predicted risk of a PSA relapse after surgery for the CAPRA score, and the CAPRA & PDE4D7 combination model. The analysis demonstrated that the two models showed better net benefit compared to the “treat all” strategy while the combined CAPRA & PDE4D7 score revealed the best net benefit across all modeled decision thresholds (0-50%; Figure 3(a)). Similarly, the net reduction analysis in primary treatment based on the two tested decision models revealed a substantial difference in treatment reduction between the CARPA score and the CARPA & PDE4D7 combination model with decision thresholds ≤30% (Figure 3(b)). At higher decision thresholds above 30% the CAPRA score might perform equivalent to the CAPRA & PDE4D7 score. However, it seems questionable whether such thresholds of 30% and higher risk of disease recurrence and progression are of any practical relevance for a patient to avoid a primary treatment like surgery.

We have illustrated that a predictive model of the clinical risk algorithm CAPRA with the prostate cancer biomarker PDE4D7 in a diagnostic needle biopsy sample provides value to risk stratification. We have demonstrated in multiple analyses that this risk prediction model performs better in stratifying prostate cancer patients to treatment relevant risk categories compared to risk schemas solely based on clinical parameters.

## 4. Discussion

Recently, we have reported the association of the PDE4D7 score to postsurgical disease recurrence in a radical prostatectomy (RP) patient cohort. Here we aimed to demonstrate that a logistic regression model developed on the same RP cohort using the preclinical risk score CAPRA with the PDE4D7 score remains its prognostic power in diagnostic needle biopsy (DB) patients. Thus, we were able to confirm the associated hazard ratio (~0.5 per score unit change) in this DB cohort to predict biochemical failure after surgery in the same order of magnitude as shown on surgical tumor tissue. We conclude that the associated hazard of the PDE4D7 score is stable across independent patient cohorts as well as in different tissue collection settings (diagnostic biopsies versus surgical resections).

Treatment decisions in primary, localized prostate cancer are largely subject to a combination of the risk of future disease progression and life expectancy. The National Comprehensive Cancer Network (NCCN) has defined six risk categories based on pretreatment clinical variables [3]. Depending on the clinical risk various options of interventions are presented in the guidelines. Multiple tools of clinical risk prediction have been developed in the form of mathematical models, which combine the value of clinical variables into a single score [25]. One of the most extensively validated clinical risk algorithms for pretreatment decision support is the CAPRA score [21]. The score is a combination of clinically available information. Originally reported in 2005, this score has been validated in several studies since then [23]. Our data illustrate that the PDE4D7 score adds important prognostic value to clinical prediction models, such as the CAPRA score, for disease-specific outcomes to support treatment decision-making.

Recently, the long-term results of the active surveillance cohort within the Göteborg randomized prostate cancer screening trial were published [26]. This data indicated that men with clinically low risk disease may have a considerable risk to experience progressive disease under a deferred treatment regime. Therefore, the authors questioned whether men other than those with very low risk disease would be eligible for expectant management strategies. The recent publication of the 10-year outcomes of the ProtecT study indicates similar conclusions in the active monitoring arm of the trial [27]. Although there is some debate about the validity of these results to contemporary practice [28] they suggest that only patients with the very lowest risk are safe of any progression during deferred treatment management. While the use of clinical criteria like CAPRA allow the selection of such a low risk patient cohort the addition of molecular markers can be expected to enlarge this very low risk patient group or further reduce the risk within this cohort. In fact, our combined CAPRA & PDE4D7 regression model defines a very low risk cohort of 22 out of 151 patients (14.6%) in the DB patient cohort with a progression-free survival (PFS) of 100% over a follow-up period of more than 15 years compared to a PFS of 85.6% for the CAPRA score alone.

Active surveillance (AS) has been established as a safe treatment alternative for men with low risk prostate cancer over the last years [29]. However, the challenge associated with AS relates to the strict monitoring schedules that men are advised to follow in order not to miss signs of progressive disease. These include the identification of elevations in PSA and upgrading of biopsy Gleason scores, which are typically "protocol triggers" to switch from AS to active treatment. Longitudinal AS studies have published decreasing patient compliance to the monitoring protocols in AS over time in particular when it comes to additional biopsy procedures [30, 31]. This issue might be addressed with a selection algorithm such as that proposed here, i.e., the combination of the CAPRA score with the prognostic genomic biomarker PDE4D7, in order to define a patient cohort with virtually no risk of future disease progression. Although this finding has to be confirmed in an AS setting, this might provide a way forward to include men into active surveillance on the basis of very limited (or no) follow-up for a given time period after the initiation of AS.

### 4.1. Limitations

A potential limitation of this study might be the sample size of the diagnostic biopsy cohort used for the CAPRA & PDE4D7 model validation. However, the measured endpoints of BCR and of secondary treatment start provide a relevant number of events (36.4% and 19.9%, respectively) for validation testing. Nevertheless, we appreciate the fact that further validation of this model is required which is warranted by the so far presented results. Another limitation might be in the selection of BCR (biochemical recurrence) as a tested endpoint. The follow-up of the diagnostic biopsy cohort was too limited to enable the testing for clinical recurrence or prostate cancer death. However, to our opinion, BCR is still a treatment relevant endpoint and is used as a decision point to a start secondary treatment in a majority of the patients (e.g., in 73.2% of patients in the RP cohort who experienced BCR a secondary treatment was started). Furthermore, PSA progression is also a typical protocol-based trigger to stop active surveillance and initiate an active intervention. Ultimately, we believe that it is important to understand the risk to experience this endpoint for an optimal treatment decision.

## 5. Conclusions 

With the data presented here we have further validated the prognostic power of the prostate cancer biomarker PDE4D7. We have demonstrated that the PDE4D7 score has equivalent performance to predict longitudinal prostate cancer outcomes when tested on tumor tissue of a diagnostic biopsy sample compared to a cancer sample that was collected after prostate resection during surgery. This study illustrates the potential of PDE4D7 for clinical use to improve presurgical risk stratification in conjunction with the routinely used prognostic clinical risk models.

## Figures and Tables

**Table 1:** Aggregated summary of the characteristics of the studied patient cohorts.** (A)** Demographics of the radical prostatectomy (RP) patient cohort including the 503 patients eligible for statistical data analysis. For patient age, preoperative PSA, percentage of tumor in biopsy, prostate volume, and PSA density the min and max values in the cohort are shown; median and IQR are depicted in parentheses. Pre- and postsurgical pathology are given (note: extracapsular extension was derived from pathology stage information). The outcome category illustrates the cumulative 5- and 10-year biochemical recurrence (BCR) and clinical recurrence to metastases (CR) postsurgical primary treatment. The treatment category lists the cumulative 5- and 10-year start to SRT (salvage radiation therapy) or SADT (salvage androgen deprivation therapy) after surgery. Mortality is shown as prostate cancer specific survival (PCSS) as well as overall survival (OS). **(B) **Demographics of the diagnostic biopsy (DB) patient cohort. In total diagnostic needle biopsy tissues of 151 were eligible for statistical data analysis. The demographics and clinical data of this cohort are presented equivalent to the RP cohort (N/A=not available).

	**Parameter**	**(A) RP cohort (n=503)**	**(B) DB cohort (n=151)**
**Demographic & Clinical **Range (median; IQR)	Age range (at RP)	41.3-74.5 (62.6; 7.4)	47.4-77.4 (64.9; 8.5)
	Preoperative PSA range	0.18-73.16 (6.7; 5.5)	2.0-49.1 (8.1; 5.7)
	Percent tumor in biopsy range	0.2-79.7 (10.3; 16.0)	N/A
	Prostate Volume range	9-148 (42; 22.5)	13.6-148.0 (38.5; 19.2)
	PSA density range	0.01-2.03 (0.16; 0.14)	0.03-1.6 (0.2; 0.17)

**CAPRA Risk Category—**No. of patients (percentage)	Low Risk (NCCN)	211 (41.9%)	38 (25.2%)
	Intermediate Risk (NCCN)	248 (49.3%)	82(54.3%)
	High Risk (NCCN)	44 (8.7%)	31 (20.5%)

**Pre-Surgery Pathology—Number of patients (percentage)**	Biopsy Gleason 3+3 (GG1)	316 (62.8%)	77 (51.0%)
	Biopsy Gleason 3+4 (GG2)	149 (29.6%)	38 (25.2%)
	Biopsy Gleason 4+3 (GG3)	25 (5.0%)	20 (13.2%)
	Biopsy Gleason >=4+4 (>=GG4)	13 (2.6%)	16 (10.6%)
	cT1	342 (68%)	97 (64.2%)
	cT2	150 (29.8%)	
	cT3	11 (2.2%)	54 (35.8%)

**Post-Surgery Pathology**—No. of patients (percentage)	Pathology Gleason 3+3 (GG1)	201 (40%)	46 (30.5%)
	Pathology Gleason 3+4 (GG2)	257 (51.1%)	52 (34.4%)
	Pathology Gleason 4+3 (GG3)	41 (8.2%)	31 (20.5%)
	Pathology Gleason >=4+4 (>=GG4)	4 (0.8%)	22 (14.6%)
	pT2	331 (65.8%)	88 (58.3%)
	pT3	172 (34.2%)	63 (41.7%)
	pT4	0 (0%)	0 (0%)
	Positive Surgical Margins	120 (23.9%)	33 (21.9%)
	Capsular Status	113 (22.5%) (=T3a)	57/145 (39.3%) infiltrated; 75/145 (51.7%) penetrated
	Positive Seminal Vesicle Invasion	60 (11.9%)	20 (13.2%)
	Positive Lymph Node Invasion	5 (1%)	10 (6.6%)

**Follow-up [months] **(IQR median follow-up)	Mean	123.6	73.7
	Median	141.8	73.6

**Outcome**—No events / total patient number (percentage; median follow-up; IQR)	BCR within 5 years	92/446 (20.6%; 121.2; 87.5)	49/151 (32.5%; 73.7; 43.9)
	BCR within 10 years	134/347 (38.6%; 134.0; 95.6)	N/A
	CR within 5 years	5/441 (1.1%; 144.4; 37.8)	4/151 (2.6%; 73.7; 42.6)
	CR within 10 years	13/306 (4.2%; 154.7; 32.85)	N/A

**Salvage Therapy**—No events / total patient number (percentage; median follow-up; IQR)	SRT within 5 years	53/439 (12.1%; 120.4; 53.5)	12/151 (7.9%; N/A; N/A)
	SRT within 10 years	83/320 (25.9%; 132.3; 39.6)	N/A
	SADT within 5 years	27/441 (6.1%; 120.7; 46.6)	16/151 (10.6%; N/A; N/A)
	SADT within 10 years	54/312 (17.3%; 132.4; 24.2)	N/A

**Survival**—No events / total patient number (percentage; median follow-up; IQR)	PCSS within 5 years	17/453 (1.1%; 144.4; 37.7)	1/151 (0.7%; N/A; N/A)
	PCSS within 10 years	38/330 (2.6%; 154.8; 30.3)	0/151 (0%; N/A; N/A)
	OS within 5 years	5/441 (3.7%; 144.4; 45.1)	1/151 (0.7%; N/A; N/A)
	OS within 10 years	10/302 (11.2%; 146.0; 35.4)	5/151 (3.3%; N/A; N/A)

**(a):** 

**Pre-Surgical Clinical Parameters**	**univariate**			**multivariate**		
**Endpoint BCR (#148/#503; 29.4**%**)**	**p value**	**HR**	**95**%** CI of HR**	**p value**	**HR**	**95**%** CI of HR**

Age at surgery	0,88	1,0	0.97 -1.03	0,77	1,00	0.96 to 1.02

Preoperative PSA	0,0002	1,03	1.01-1.04	0,00	1,08	1.03 to 1.1

PSA density	0,0100	2,15	1.2 to 3.8	0,03	0,12	0.02 to 0.8

*Biopsy Gleason Score 3+3 (N=316); reference*						

Biopsy Gleason Score 3+4 (N=149)	0,0003	1,9	1.4-2.8	0,02	1,56	1.06 to 2.3

Biopsy Gleason Score >=4+3 (N=38)	<0.0001	6,2	3.9-9.7	<0.0001	4,82	2.9 to 7.9

Percentage Positive Biopsy Cores	<0.0001	4,2	2.3-7.7	0,08	2,29	0.91 to 5.7

Percentage Tumor in Biopsy	<0.0001	1,0	1.02-1.04	0,00	1,02	1.01 to 1.04

*Clinical stage cT1c (N= 342); reference*						

Clinical stages cT2 & cT3 (N=161)	<0.0001	2,1	1.5 to 2.9	0,20	1,27	0.88 to 1.8

PDE4D7 (continuous)	<0.0001	0,53	0.41 to 0.67	<0.0001	0,52	0.4 to 0.68

**Pre-Surgical Clinical Parameters**	**univariate**			**multivariate**		

**Endpoint BCR (#148/#503; 29.4**%**)**	**p value**	**HR**	**95**%** CI of HR**	**p value**	**HR**	**95**%** CI of HR**

CAPRA Score	<0.0001	1,5	1.3 to 1.6	<0.0001	1,7	1.5 to 1.9

PDE4D7 (continuous)	<0.0001	0,47	0.33 to 0.65	<0.0001	0,56	0.43 to 0.72

**(b):** 

**Pre-Surgical Clinical Parameters**	**univariate**			**multivariate**		
**Endpoint BCR (#55/#151; 36.4**%**)**	**p value**	**HR**	**95**%** CI of HR**	**p value**	**HR**	**95**%** CI of HR**

Age at surgery	0,81	0,99	0.95 to 1.04	0,006	0,94	0.89 to 0.98

Preoperative PSA	0,0001	1,04	1.02 to 1.06	0,002	1,1	1.03 to 1.2

PSA Density	0,003	2,7	1.4 to 5.3	0,18	0,26	0.04 to 1.9

*Biopsy Gleason GG1 (n=85); reference*						

Biopsy Gleason GG2 (n=44)	0,07	1,9	0.95 to 3.8	0,10	1,9	0.89 to 3.9

Biopsy Gleason GG3 (n=20)	0,0001	4,3	2.04 to 9.2	0,02	2,6	1.2 to 5.7

Biopsy Gleason GG4 (n=7)	0,0007	5,7	2.1 to 15.8	0,01	4,1	1.4 to 11.6

Biopsy Gleason GG5 (n=11)	<0.0001	8,0	3.5 to 18.4	0,03	2,9	1.1 to 7.6

Percentage Positive Biopsy Cores	0,013	4,6	1.4 to 15.2	0,19	2,4	0.63 to 9.4

*Clinical stage cT2 (n= 108); reference*						

Clinical stage cT3 (n=60)	0,007	2,0	1.2 to 3.4	0,05	1,7	0.99 to 3.1

PDE4D7 (continuous)	<0.0001	0,47	0.33 to 0.65	<0.0001	0,43	0.29 to 0.63

**Pre-Surgical Clinical Parameters**	**univariate**			**multivariate**		

**Endpoint BCR (#55/#151; 36.4**%**)**	**p value**	**HR**	**95**%** CI of HR**	**p value**	**HR**	**95**%** CI of HR**

CAPRA Score	<0.0001	1,5	1.3 to 1.6	<0.0001	1,4	1.2 to 1.6

PDE4D7 (continuous)	<0.0001	0,47	0.33 to 0.65	0,0001	0,53	0.38 to 0.74

## Data Availability

The data used to support the findings of this study are currently under embargo while the research findings are commercialized. Requests for data, 12 months after publication of this article, will be considered by the corresponding author.

## Abbreviations

AS: Active surveillance
RP: Radical prostatectomy
DB: Diagnostic biopsy
BCR: Biochemical recurrence
STFS: Secondary treatment free survival
HR: Hazard ratio
CI: Confidence interval
NPV: Negative predictive value
AUC: Area under the curve
DCA: Decision curve analysis
CAPRA score: Cancer of the Prostate Risk Assessment score.

## Supplementary Materials

Supplementary Materials
** Supplementary Figure** **1A: **overview of the statistics and distribution of the normalized PDE4D7 RT-qPCR values.** Supplementary Figure ****1B: **summary statistics of the reference gene normalized and transformed PDE4D7 score.** Supplementary Figure ****2: **data analysis design of the generated PDE4D7 score data in the radical prostatectomy (RP) cohort.** Supplementary Figure ****3A: **study design of the radical prostatectomy (RP) cohort.** Supplementary Figure ****3B:** study design of the diagnostic biopsy (DB) cohort.** Supplementary Figure ****4A: **Kaplan-Meier survival analysis of the time to PSA relapse after radical prostatectomy in the Taylor patient cohort (n=130) [20]; Kaplan-Meier analysis of the biochemical recurrence (BCR) free survival of the CAPRA*∗* score categories.** Supplementary Figure ****4B: **Kaplan-Meier survival analysis of the time to PSA relapse after radical prostatectomy in the Taylor patient cohort (n=130) [20]; Kaplan-Meier analysis of the CAPRA*∗*  & PDE4D7 score combination model.
