# Supplementary material for: The Prognostic PDE4D7 Score in a Diagnostic Biopsy Prostate Cancer Patient Cohort with Longitudinal Biological Outcomes
Source: Prostate Cancer. 2018 Jul 26;2018:5821616. doi: 10.1155/2018/5821616 (PMC6083737; doi:10.1155/2018/5821616)
Supplement: Supplementary Materials — Supplementary Figure 1A: overview of the statistics and distribution of the normalized PDE4D7 RT-qPCR values. Supplementary Figure 1B: summary statistics of the reference gene normalized and transformed PDE4D7 score. Supplementary Figure 2: data analysis design of the generated PDE4D7 score data in the radical prostatectomy (RP) cohort. Supplementary Figure 3A: study design of the radical prostatectomy (RP) cohort. Supplementary Figure 3B: study design of the diagnostic biopsy (DB) cohort. Supplementary Figure 4A: Kaplan-Meier survival analysis of the time to PSA relapse after radical prostatectomy in the Taylor patient cohort (n=130) [20]; Kaplan-Meier analysis of the biochemical recurrence (BCR) free survival of the CAPRA∗ score categories. Supplementary Figure 4B: Kaplan-Meier survival analysis of the time to PSA relapse after radical prostatectomy in the Taylor patient cohort (n=130) [20]; Kaplan-Meier analysis of the CAPRA∗ & PDE4D7 score combination model. [file 5821616.f1.pdf]

Supplementary Materials

Supplementary Figure 1A

PDE4D7_norm Distribution

Summary Statistics

Sample size	151
Lowest value	-5.62
Highest value	4.36
Arithmetic mean	-0.6279
95% CI for the Arithmetic mean	-0.9369 to -0.3190
Median	-0.41
95% CI for the median	-0.8216 to -0.09907
Variance	3.6908
Standard deviation	1.9211
Relative standard deviation	-3.0594 (-305.94%)
Standard error of the mean	0.1563
Coefficient of Skewness	-0.3020 (P=0.1241)
Coefficient of Kurtosis	-0.06317 (P=0.9935)
D'Agostino-Pearson test for Normal distribution	accept Normality (P=0.3065)
**Percentiles**	**95% Confidence interval**
2.5 (-4.4842)	-5.6200 to -4.1316
5 (-4.139)	-4.7320 to -3.7012
10 (-3.372)	-4.1241 to -2.6899
25 (-1.7775)	-2.4117 to -1.2900
75 (0.7425)	0.3075 to 0.9983
90 (1.554)	1.2366 to 2.2564
95 (2.3535)	1.7429 to 2.8653
97.5 (2.8217)	2.3056 to 4.3024

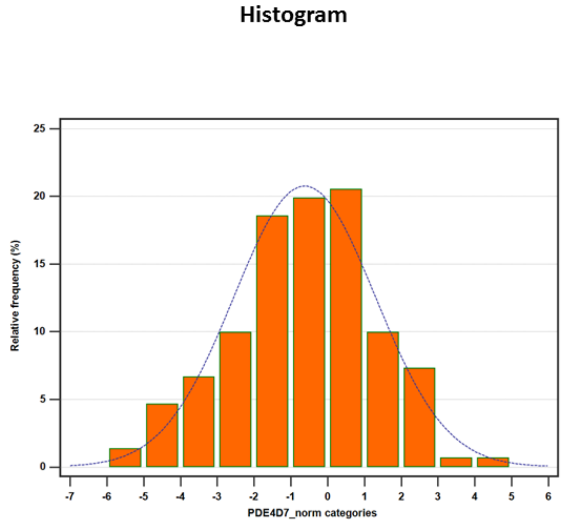

Supplementary Figure 1A. Overview of the statistics and distribution of the normalized PDE4D7 RT-qPCR values. **(A)** Summary statistics of the reference gene normalized PDE4D7 distribution. The qPCR values were normalized to four reference genes as described in the methods section. The table outlines the summary statistics while the histogram provides an overview of the distribution of the quantitative values. **(B)** Summary statistics of the reference gene normalized and transformed PDE4D7 score. The normalized PDE4D7 values were linearly transformed to a 1-5 distribution. The table outlines the summary statistics while the histogram provides an overview of the distribution of the quantitative values.

Supplementary Figure 1B

PDE4D7 Score Distribution

Summary Statistics

Sample size	151
Lowest value	1
Highest value	5
Arithmetic mean	3.0028
95% CI for the Arithmetic mean	2.8790 to 3.1266
Median	3.09
95% CI for the median	2.9293 to 3.2105
Variance	0.5928
Standard deviation	0.7699
Relative standard deviation	0.2564 (25.64%)
Standard error of the mean	0.06266
Coefficient of Skewness	-0.3030 (P=0.1228)
Coefficient of Kurtosis	-0.05919 (P=0.9976)
D'Agostino-Pearson test for Normal distribution	accept Normality (P=0.3040)
**Percentiles**	**95% Confidence interval**
2.5 (1.4538)	1.0000 to 1.6000
5 (1.6)	1.3552 to 1.7709
10 (1.906)	1.6000 to 2.1751
25 (2.54)	2.2933 to 2.7400
75 (3.55)	3.3821 to 3.6500
90 (3.878)	3.7483 to 4.1601
95 (4.1975)	3.9485 to 4.4041
97.5 (4.3872)	4.1791 to 4.9770

Histogram

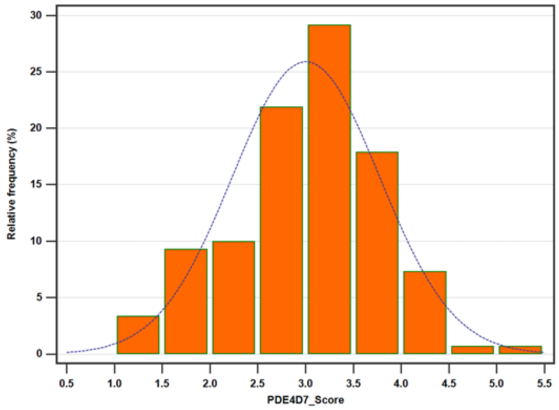

Supplementary Figure 1B. Summary statistics of the reference gene normalized and transformed PDE4D7 score. The normalized PDE4D7 values were linearly transformed to a 1-5 distribution. The table outlines the summary statistics while the histogram provides an overview of the distribution of the quantitative values.

Supplementary Figure 2

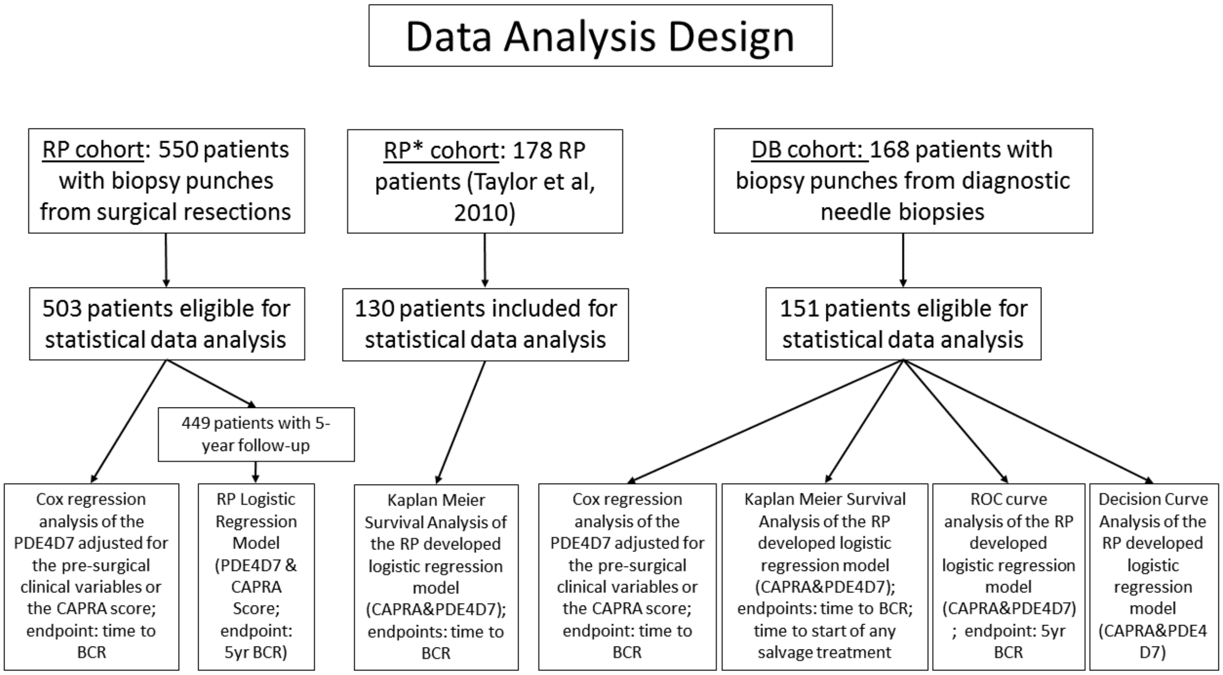

Supplementary Figure 2. Analysis design of the generated PDE4D7 score data in the two patient study cohorts. The surgery cohort (RP) included 550 patients of which 503 were eligible for statistical data analysis after quality control and after removal of patients with adjuvant and neo-adjuvant hormone therapy before or after surgery. The Taylor cohort (Taylor et al, 2010) consisted of 178 patients of which 130 patients were included into this study. The diagnostic biopsy cohort comprised 168 patients of which 151 were eligible for statistical data analysis after quality control. The respective data analysis procedures are outlined. Note: the CAPRA & PDE4D7 combination model was developed and fixed by logistic regression on the RP cohort. This set model was used for testing and validation on the DB cohort.

Supplementary Figure 3A

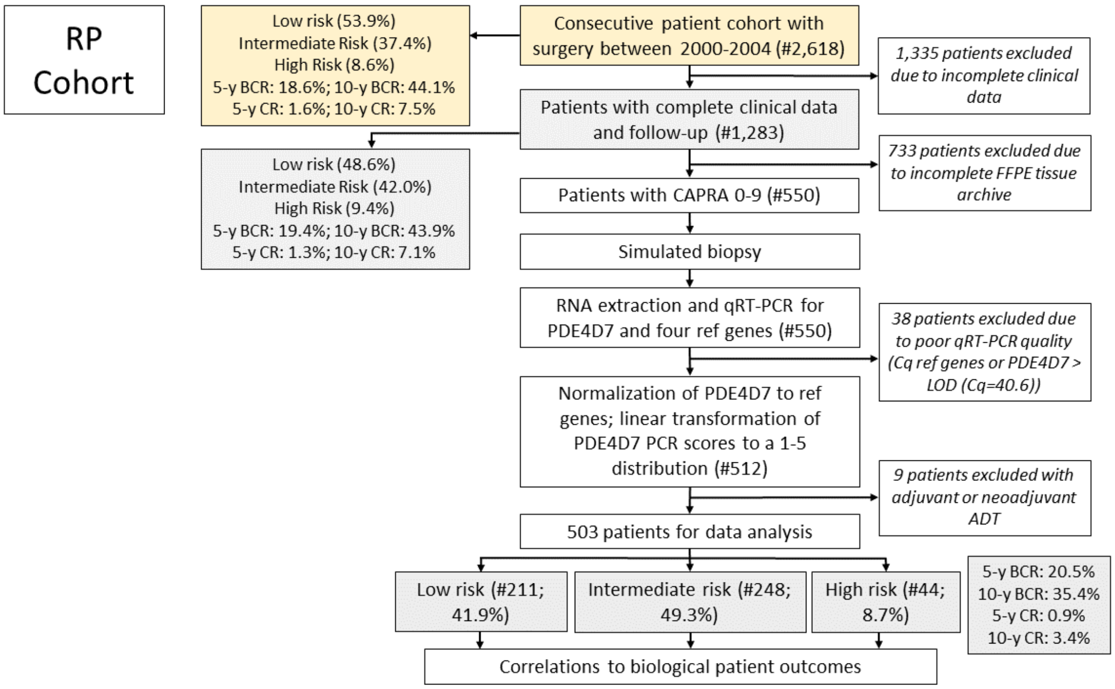

Supplementary Figure 3A. Summary overview of the design of the two patient study cohorts. **(A)** Design of the radical prostatectomy (RP) cohort. Patients were operated at the Martini Klinik, Hamburg, Germany between 2000-2004. **(B)** Design of the radical prostatectomy (RP) cohort. Patients were operated at the University Hospital Muenster, Germany between 1994-2011. The risk stratification of the patients were performed according to the D'Amico risk criteria. BCR – biochemical recurrence after surgery; CR – clinical recurrence to metastases after surgery.

Supplementary Figure 3B

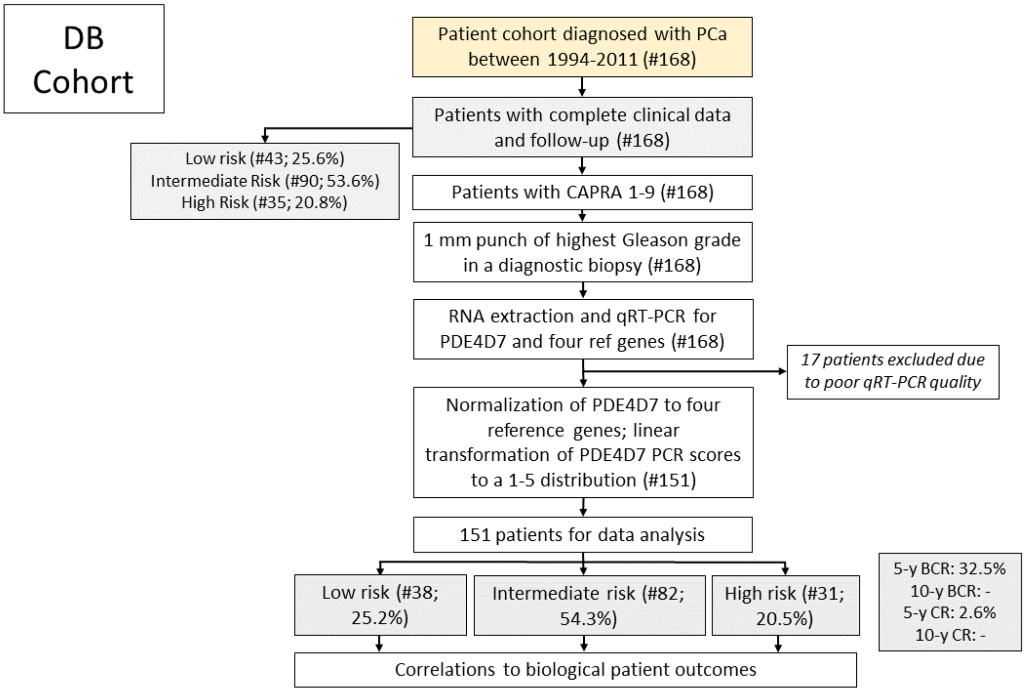

Supplementary Figure 3B. Design of the radical prostatectomy (RP) cohort. Patients were operated at the University Hospital Muenster, Germany between 1994-2011. The risk stratification of the patients were performed according to the D'Amico risk criteria. BCR – biochemical recurrence after surgery; CR – clinical recurrence to metastases after surgery.

Supplementary Figure 4A

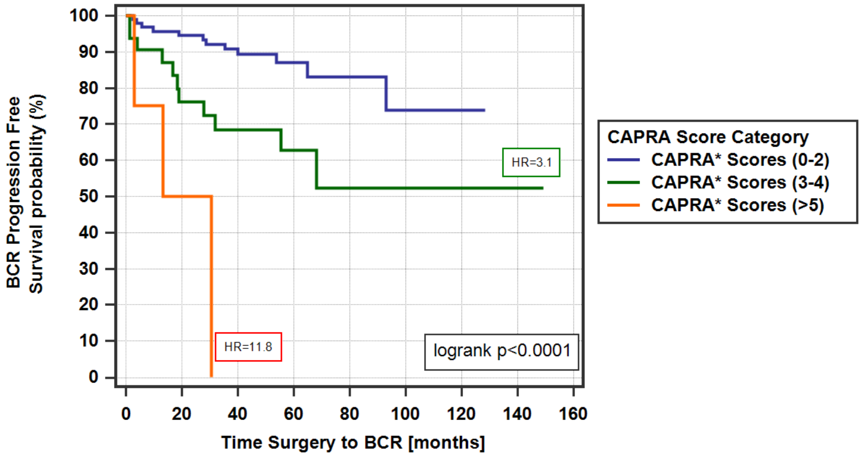

Number at risk

Group	0	20	40	60	80	100	120	140	160
Group: CAPRA* Scores (0-2)	94	83	61	29	15	7	2	0	0
Group: CAPRA* Scores (3-4)	32	21	17	8	3	2	1	1	0
Group: CAPRA* Scores (>5)	4	2	0	0	0	0	0	0	0

Supplementary Figure 4B

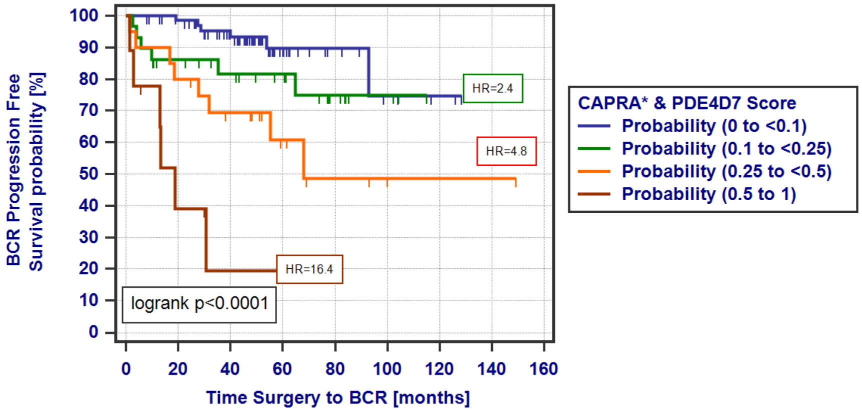

Number at risk

Group	0	20	40	60	80	100	120	140	160
Group: Probability (0 to <0.1)	72	65	47	17	8	4	2	0	0
Group: Probability (0.1 to <0.25)	29	22	18	14	7	3	0	0	0
Group: Probability (0.25 to <0.5)	20	16	12	6	3	2	1	1	0
Group: Probability (0.5 to 1)	9	3	1	0	0	0	0	0	0

Supplementary Figure 4. Kaplan Meier survival analysis of the time to PSA relapse after radical prostatectomy in the Taylor patient cohort (n=130). **(A)** Kaplan-Meier analysis of the biochemical recurrence (BCR) free survival of the CAPRA* score

categories, and **(B)** the CAPRA* & PDE4D7 score combination model; the combination model was developed by logistic regression of the CAPRA* and the PD4D7 scores in the RP patient cohort (n=503) and used as such for testing in the Taylor patient cohort. The logit(p) function was transformed to p=1/(1+e^(-logit(p)) in order to calculate the probability p for an individual patient to experience a biochemical relapse within 5 years after surgery. Censored patients are indicated by vertical bars. The Hazard Ratio's (HR) compared to the reference group (indicated in blue) and the logrank p-values are provided. Number of men at risk are given in the table below the Kaplan Meier survival graph. The CAPRA* score is calculated based on Cooperberg et al, 2005; however, as the information on the number of positive biopsy cores was missing the CAPRA* score was calculated using patient age, pre-operative PSA, biopsy Gleason score, and clinical stage. The influence of the missing information on the biopsy cores was very limited in the RP as well as the DB cohort (data not shown). The CAPRA* score categories are defined as: CAPRA* (1): CAPRA scores 0-2; CAPRA* (2): CAPRA scores 3-5; CAPRA* (3): CAPRA scores ≥6. The lowest CAPRA* score category (1) was used as the reference category. The CAPRA* & PDE4D7 score categories are defined according to the probability to experience PSA failure after surgery based on the logit(p) function of the logistic regression model. The categories are indicated.
